# Fast-Track and Integration-Free Method of Genome Editing by CRISPR/Cas9 in Murine Pluripotent Stem Cells

**DOI:** 10.3389/fcell.2022.819906

**Published:** 2022-03-18

**Authors:** Md Mahfuz Al Mamun, Ihtisham Bukhari

**Affiliations:** ^1^ Translational Research Institute, Henan Provincial People’s Hospital, Henan Key Laboratory of Stem Cell Differentiation and Modification, School of Clinical Medicine, Henan University, Zhengzhou, China; ^2^ Henan Key Laboratory of Helicobacter pylori, Microbiota and Gastrointestinal Cancer, Marshall Medical Research Center, Department of Gastroenterology, Fifth Affiliated Hospital of Zhengzhou University, Zhengzhou, China

**Keywords:** CRISPR/Cas9-mediated genome editing, genome editing, embryonic stem, gene knockout, protocol

## Abstract

The CRISPR/Cas9 system has unprecedentedly revolutionized genome-editing technology, which is being successfully applied virtually in all branches of biological sciences. Although much success has been attained in gene manipulation, still the majority of methods are laborious and non-integration-free, and require prolonged time for the expansion of mutant cell pools/clones, while fewer cells exhibit functional knockout efficiency. To overcome these obstacles, here, we describe an efficient, inexpensive, integration-free, and rapid one-step protocol for CRISPR/Cas9-assisted gene knockout in murine pluripotent stem cells (PSCs). Our protocol has streamlined both the liposome-based transfection system and screening strategy to work more efficiently with small numbers of PSCs (∼2.0 × 10^4^ cells) and to minimize laborious steps of lentiviral packaging, transduction, and single-clone passaging. In our method, around 90% (CI = 95%, 79.5230%–100%) of PSC colonies harbored functional knockout in the context of protein expression. Therefore, the current protocol is technically feasible, time-saving, and highly efficient for genome editing in pluripotent stem cells.

## Graphical Abstract

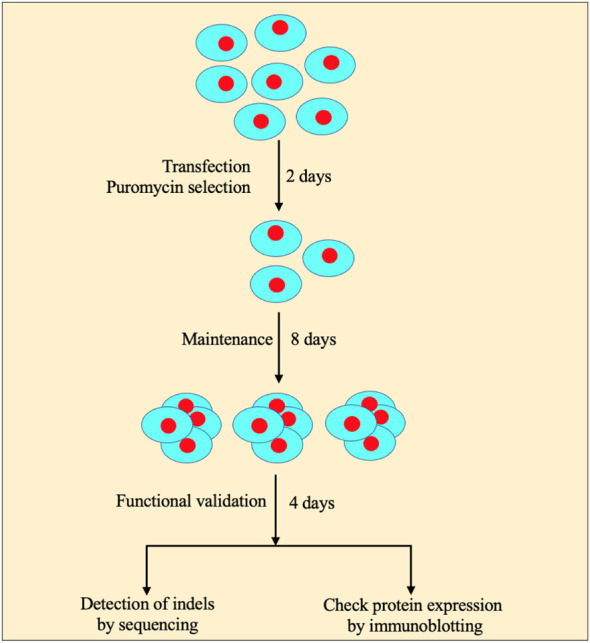

## Introduction

The discovery of CRISPR (clustered, regularly interspaced, short palindromic repeats)/Cas9 (CRISPR-associated system protein 9)-mediated genome-editing technology has revolutionized the field of genetic engineering (Lino et al., 2018; Li et al., 2020). The feasibility and flexibility of the CRISPR/Cas9 system in the context of site-specific endonuclease activity on the genome have turned it into a leading “genome editing tool” for biological studies (Sanjana et al., 2014; Lino et al., 2018). In this system, Cas9 is guided to a specific locus of the target genome by a short 20-nucleotide sequence referred to as guider RNA (gRNA) that lies on the upstream region of the NGG PAM (protospacer-adjacent motif) (Cao et al., 2010; Jinek et al., 2012; Mali et al., 2013; Shalem et al., 2014). Cas9 introduces double-stranded DNA breaks after three nucleotides at the 5′ end of NGG PAM (Jinek et al., 2012; Mali et al., 2013). The double-stranded DNA breaks in eukaryotic organisms are generally repaired *via* the error-prone non-homologous end-joining mechanism that frequently generates small indels (insertion–deletion mutations) at the Cas9-targeted genomic site, resulting in a permanent mutation in protein-coding genes (Mao et al., 2008; Mali et al., 2013; Sternberg et al., 2014).

Growing evidence suggests that a substantial number of gene knockout experiments using CRISPR/Cas9 containing vector plasmids such as pLentiCRISPR V2 (single vector) or separately expressed pLentiCas9 plus pLentiGuide-Puro (two-vector system) (Sanjana et al., 2014; Shalem et al., 2014) have failed. Different factors are responsible for the aforementioned failure, such as low titers of lentiviral particles during infection, selection of gRNAs targeting multiple sites on a genome at the same time, selective markers working ineffectively for constitutive expression of the transgene Cas9, hard-to-transfect cells such as pluripotent stem cells (e.g., embryonic or induced pluripotent stem cells or primary cells), low transduction efficiency of lentiviruses depending on the cell types (e.g., embryonic or induced pluripotent stem cells or primary cells), and overgrowth of wild type or wild-type-like clones during the selection of mutant cell clones. Although the maximal transduction efficiency in PSCs is gained through the lentiviral gene delivery system estimating around 20% with over 95% cell viability (Cao et al., 2010), this requires prolonged time for clonal expansion of edited cell pools as single cells into 96-well plates, which is laborious. It is also frequently associated with the poor quality of the target cells (Vicente et al., 2021). Prolonged time for the selection of mutant cell pools with selectable markers (e.g., puromycin) in some cases may also result in the off-target effects during CRISPR/Cas9 assisted gene editing (Vicente et al., 2021). Additionally, in lentiviral systems, the transgenes, for example, puromycin and Cas9, are integrated into the genome of the host, indicating a key drawback of the majority of genome-editing tools, which is a debatable issue for safety in clinical trials (Chakraborty, 2019; Vicente et al., 2021). However, direct DNA delivery (e.g., CRISPR/Cas9) by electroporation into PSCs is associated with poor cell survival with over 60% cell death (Cao et al., 2010). To overcome these obstacles and challenges, here, we report a single-step, rapid, and integration-free liposome-based (lipofectamine) modified methodology of CRISPR/Cas9 mediated genome editing in PSCs, which effectively works either with lenti/non-lenti CRISPR backbones having different selectable markers.

## Step-by-Step Protocol Details

### Reagent Formulation

#### Key Reagents and Resources

A list of key reagents and resources is given in the Supplementary Material (Supplementary Table S1).

#### Formulation of the LB Medium and Agar Plate

Detailed formulation of the respective LB medium and agar plate is given in Supplementary Material (Supplementary Protocol Section, 1.1) (Supplementary Table S2).

#### Formulation of the Mouse Pluripotent Stem Cell Medium

**Table:** 

Reagent	Stock concentration	Volume to add
DMEM (high glucose)	NA	500 ml
GlutaMAX™ supplement	100X	6 ml
KnockOut™ serum replacement	NA	50 ml
Embryonic stem-cell FBS	NA	50 ml
2-Mercaptoethanol*	NA	6 ml
Penicillin–streptomycin	10,000 U/mL	6 ml
MEM non-essential amino acids solution	100X	6 ml
Sodium pyruvate	100 mM	6 ml
ESGRO^®^ recombinant mouse LIF protein**	10^6^ units/ml	5.8 per 50 ml

Note: Use a 500 ml 0.2 μM low protein-binding filter flask to sterilize the medium after adding all components. Test an aliquot in a 37 °C incubator to ensure that the medium is sterile.

*2-Mercaptoethanol should be molecular cell culture grade (Sigma, Cat# M3148). To prepare stock: add 70 μl 2-mercaptoethanol + 100 ml DPBS.

**Add 5.8 μl Mouse LIF to 50 ml medium just before use.

#### Preparation of the 0.1% gelatin-coated plate

i. Add appropriate amounts of 0.1% gelatin solution as below:

**Table:** 

Wells	Amount/well
96	100 μl
48	300 μl
24	0.5 ml
12	1.0 ml
6	1.5–2.0 ml

 ii. Incubate the plate for 30 min at room temperature
iii. Remove the gelatin solution
iv. Immediately add cells to the dish

**Note:** Do not completely dry the plates.

#### Culture of Mouse PSCs

Re-thaw the mouse PSCs (e.g., R1 cells, 1 × 10^8^) from stock and allow them to grow in 0.1% gelatin-coated 6-cm dish containing mouse PSC medium at 37°C and 5% CO_2_. Passage the cells at 70% confluence (around 2 days).

#### Formulation of 2X Laemili Sample Buffer

See Supplementary Material (Supplementary Table S3).

### Single-Guide RNA Design and Synthesis

**FIGURE 1:**
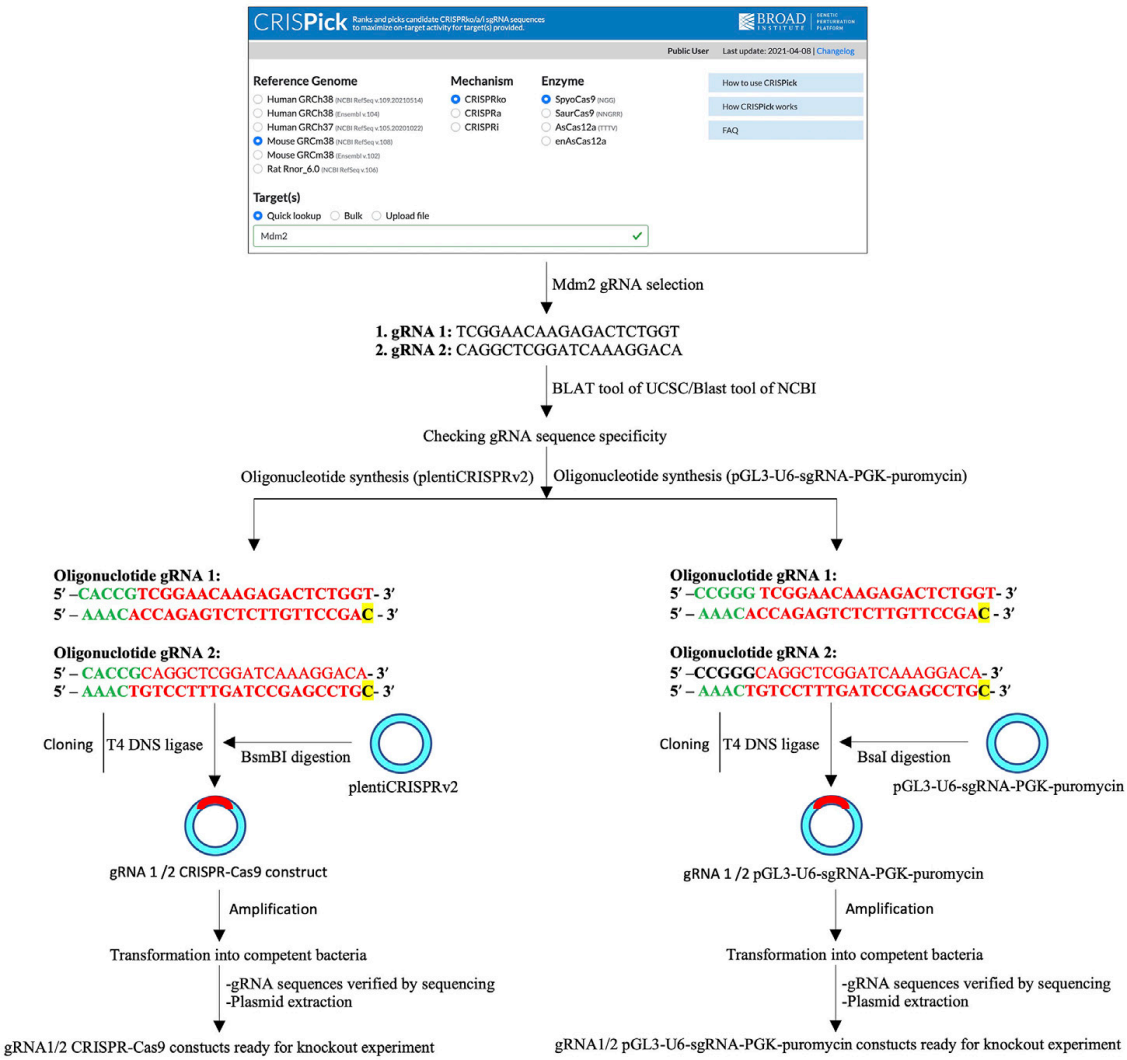
Schematic of single- and two-vector construct preparation. A schematic of the single-guide RNA (sgRNA or gRNA) design, sgRNA primer synthesis, CRISPR/Cas9 (left) or pGL3-U6-sgRNA-PGK-puromycin (right) digestion and ligation of the sgRNA insert, and bacteria-based cloning of CRISPR/Cas9-sgRNA (left) or pGL3-U6-sgRNA-PGK-puromycin-sgRNA (right) constructs.

Protocol concerning sgRNA design and synthesis is described in detail in the Supplementary Material (Supplementary Protocol Section 2.1–2.3) (Figure 1).

### Preparation of Single- and Two-Vector Constructs

See protocol details about single-vector or two-vector construction in Supplementary Material (Supplementary Protocol Section 2.4.1–2.4.3) (Figure 1).

### Transfection of Mouse Pluripotent Stem Cells

**FIGURE 2:**
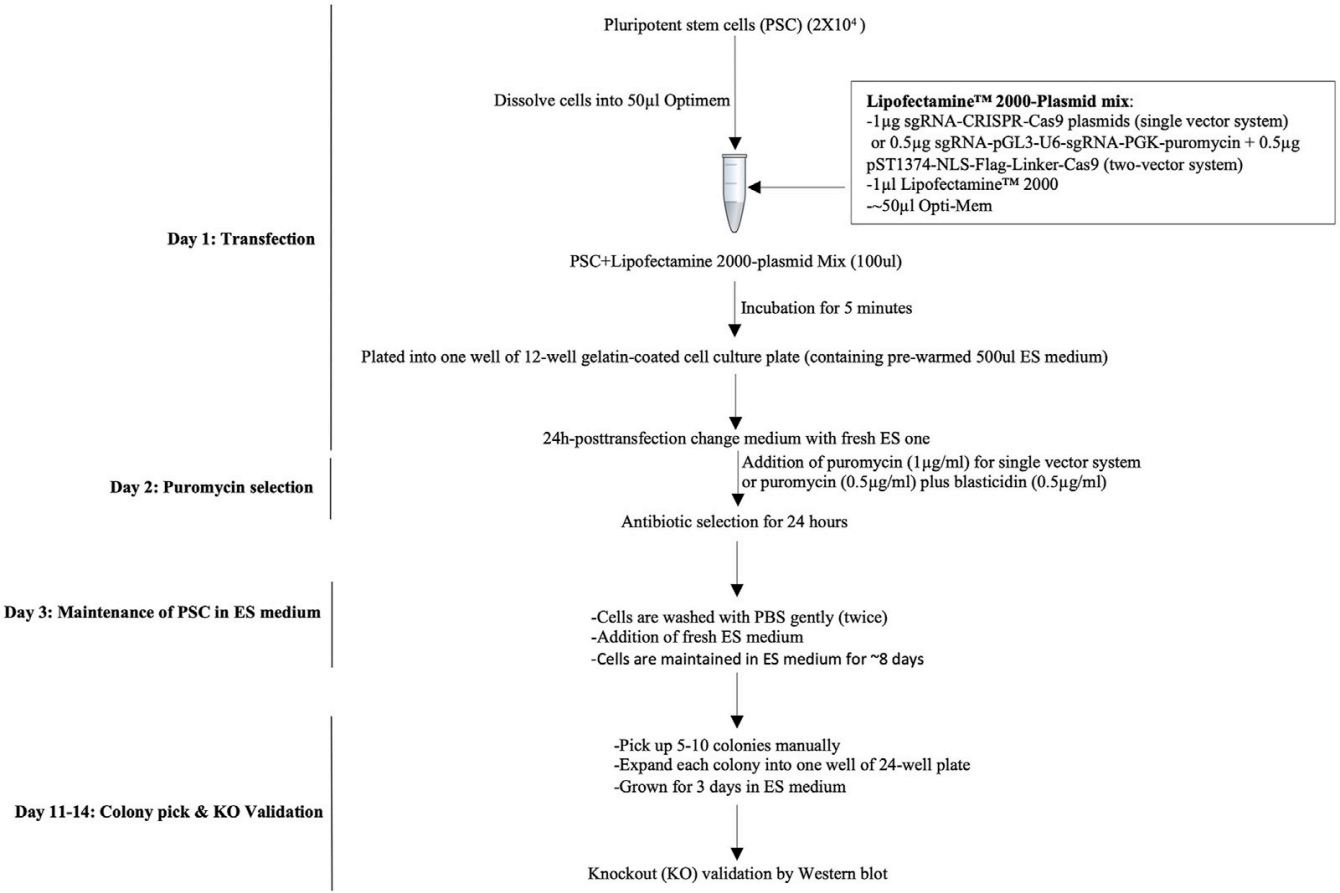
Workflow of the CRISPR/Cas9-mediated knockout strategy in pluripotent stem cells. A workflow of the CRISPR/Cas9-assisted knockout strategy consists of transfection of PSCs under the single-cell stage, quick antibiotic(s) selection, and colony pickup for sustainable growth and knockout validation.

Time: 1 day (Figure 2).

a. Preparation of Lipofectamine^R^2000-sgRNA-CRISPR/Cas9 plasmid mix or Lipofectamine^R^2000-sgRNA-pGL3-U6-sgRNA-PGK-puromycin plasmid mix was performed as follows:

For Lipofectamine^R^2000-sgRNA-CRISPR/Cas9 plasmid mix:

**Table:** 

Component	Amount	Incubation time (min)
		(Room temperature)
Lipofectamine^R^2000 + Opti-MEM^®^ medium (A)	1 μl + 25 μl	5
plentiCRISPR/Cas9 plasmid-sgRNA + Opti-MEM^®^ medium (B)	1 μg + 25 μl	5
A + B	∼50^*^	20

*Total amount might vary depending on the plasmid concentration.

For Lipofectamine^R^2000-sgRNA-pGL3-U6-sgRNA-PGK-puromycin plasmid mix.:

**Table:** 

Component	Amount	Incubation time (min)
		(Room temperature)
Lipofectamine^R^2000 + Opti-MEM^®^ medium (A)	1 μl + 25 μl	5
pGL3-U6-sgRNA-PGK-puromycin-sgRNA + pST1374-NLS-Flag-Linker-Cas9 + Opti-MEM^®^ medium (B)	0.5 μg + 0.5 μg + 25 μl	5
A + B	∼50^*^	20

*Total amount might vary depending on the plasmid concentration.

**Note**: The control group contains only empty plentiCRISPR V2 or sgRNA-pGL3-U6-sgRNA-PGK-puromycin. The control is prepared simultaneously in the same way.

b. Dissolve 2 × 10^4^ PSCs into 50 μl Opti-MEM^®^ medium, immediately add the Opti-MEM containing PSCs into the Lipofectamine^R^2000-CRISPR/Cas9-gRNA plasmid mixture (A + B) or Lipofectamine^R^2000-sgRNA-pGL3-U6-sgRNA-PGK-puromycin plasmid mixture, pipette gently to mix thoroughly (2 times), and then incubate at room temperature for 5 min.

**Note:** A total of 2 × 10^4^ cells can be increased up to 4 × 10^4^ cells under the same conditions, but it will lead to a high number of surviving clones (closely packed).

**CRITICAL**: Dissolve the PSC cells into Opti-MEM^®^ medium just before the completion of step 2.4. a.

**CRITICAL:** The incubation period of Lipofectamine^®^2000-plasmid plus PSC cell mixture should not exceed more than 5 min.

**Note:** A control lenti-CRISPR/Cas9 plasmid or pGL3-U6-sgRNA-PGK-puromycin without sgRNA can be transfected to check the effects of transfection on ES cells.

c. Add the cell-plasmid mixture from step 2.4. b into a single well of 0.1% gelatin-coated 12-well plate containing 500 μl ES medium (pre-warmed) and incubate in a cell incubator (37°C, 5% CO_2_) for 24 h.

### Antibiotic Selection

Time: 1 day.

a. Wash cells gently with PBS and add fresh 1 ml ES medium containing puromycin (1 μg/ml) for a single-vector system 24 h post-transfection. Similarly, for a two-vector system, use two antibiotics at the same time, such as puromycin (0.5 μg/ml) and blasticidin (0.5 μg/ml).

**Note:** replace Puromycin or simultaneous “puromycin plus blasticidin” selection was performed to remove non-transfected cells.

b. After 24 h of puromycin (single-vector system) or puromycin + blasticidin selection (two-vector system) (from step 2.4. a), wash the cells gently with PBS and add a fresh PSC medium.

**Note:** ∼0.1% PSC cells survived following 24 h of puromycin treatment.

### Culture and Maintenance of PSCs

Time: 7–8 days.

a. Change the medium every day with a fresh PSC medium. Cells should be maintained in an ES medium for additional 7–8 days (Figure 2).
b. PSCs form colonies (50–100 cells/colony) (step 2.6. a) that can be seen with naked eyes. Pick up 3–5 colonies (here, we picked five colonies), split, and passage every single colony into a different well (e.g., 2–3) in a 24-well plate (0.1% gelatin-coated) containing 250 ml PSC medium. Grow cells for additional 2–3 days and change the medium every other day.
c. Proceed to knockout validation.

### Knockout Validation

Time: 2–3 days.

#### Functional Validation

**Note**: In this protocol, at first, we checked if the surviving cells could express relevant genes since in certain cases, indels in genes do not affect their protein expression. Only those cells functionally defective of protein expression were considered for DNA sequencing.

a. Harvest proteins from PSCs of different groups using the 10X cell lysis buffer (CST, Cat# #9803) as per manufacturer’s instruction.

**Note**: Here, we harvested proteins from the control group as well as five experimental groups.

b. Use 2X Laemmli sample buffer for SDS-PAGE protein sample preparation (sample: 2X sample buffer = 1:1) followed by heating at 95°C for 10 min.
c. Place the sample on ice for instant use or store at −80 °C for future use.
d. Perform Western blot as described previously (Yang and Mahmood, 2012).

**FIGURE 3:**
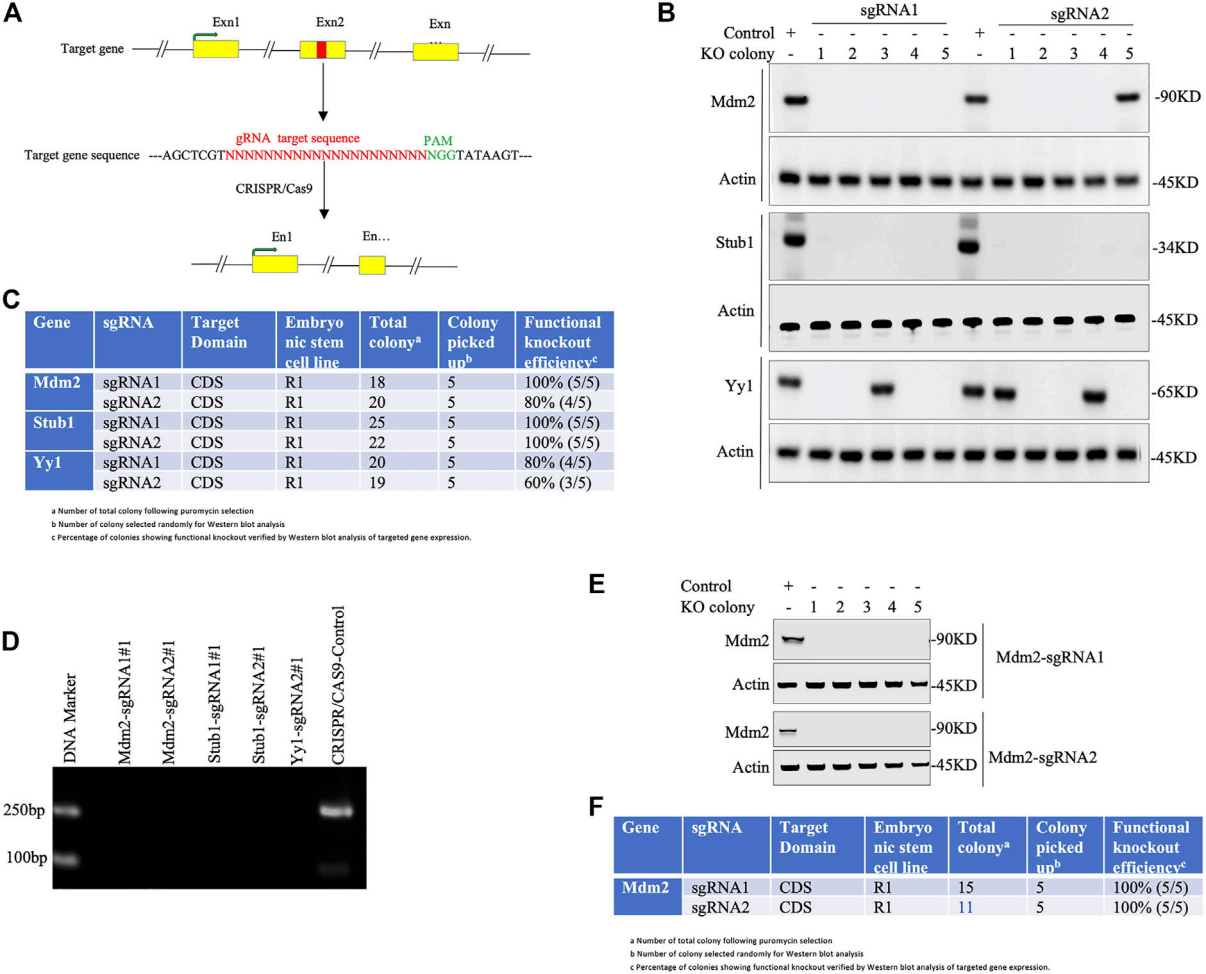
Knockout validation in pluripotent stem cells. **(A)** Simplified representation of a sgRNA targeting exon (designated as Exn1, Exn2, Exn3, … ) of a genomic DNA. Exons highlighted in yellow and red colored region represents the sgRNA-targeted sequence that lies before the PAM sequence (green). Cas9 creates double-stranded breaks on target DNA, resulting in the disruption of the Exn2 sequence. **(B-D)** Knockout validation in a single-vector system (lentiviral backbone). **(B)** Western blot analysis of knockout cells. Total five colonies of each sgRNA (numbered as 1, 2, 3, 4, and 5) were randomly selected to validate for functional knockout (KO) by immunoblotting against given antibodies. Actin is used as an internal loading control throughout. The control is a wild-type PSC. **(C)** Tabulated representation of each gene with their respective sgRNAs and colonies showing the efficiency of functional knockout (%) obtained by Western blot analysis of respective gene expression. **(D)** Gel electrophoretic images of amplicon from the PCR experiment of knockout colonies of Mdm2, Stub1, and yy1 genes (colony number #1) and the control CRISPR/Cas9 plasmid. **(E-F)** Knockout validation in a two-vector system (non-lentiviral backbone). **(E)** A total of five colonies of each sgRNA (Mdm2) were picked randomly to justify functional knockout (KO) by Western blotting using the given antibodies. Actin was used as the internal loading control. **(F)**. Western blot-based functional knockout efficiency (%) of a sgRNA targeting a specific gene.

**Note**: For functional validation of knockout, immunofluorescence or flow cytometry analysis can also be performed (Figure 3).

e. Count the functional knockout efficiency (%) of gRNA using the following formula (Figure 3):

$$\text{Functional knockout efficiency}\left(\text{\%}\right)=\frac{\text{Number of PSC colony lacking protein expression X }100}{\text{Total number of PSC colony}}.$$

#### Determination of Indel Frequency of gRNA

**Note:** The CRISPR/Cas9 system involves insertion or deletion (indel) mutations to genomic DNA which can be detected using Sanger sequencing.

a. Extract genomic DNAs from different cell groups using a TIANamp genomic DNA kit, according to manufacturer’s instruction.

**Note:** Take the control and functionally validated knockout groups only.

b. Design a pair of PCR primers for the amplification of a genomic region targeted by sgRNA that contains predicted Cas9 cut site; see a list of PCR primers in Supplementary Material (Supplementary Protocol Section 2.5).

**Note:** The recommended amplicon size is 500–600bp and design primers accordingly.

**Critical:** Same PCR primers will also be used to amplify a genomic region of control PSCs.

c. Set up PCR reaction and conditions as follows:

PCR reaction:

**Table:** 

Component	Amount
Forward primer (10 μM)	2 μl
Reverse primer (10 μM)	2 μl
2× Taq plus master mix	25 μl
Genomic DNA	500 ng
ddH_2_O	Up to 50 μl
Total	50 μl

PCR conditions:

**Table:** 

Step	Temperature (C)	Time	Cycles
Initial denaturation	94°	5 min	1
Denaturation	94°	30 s	30–35
Annealing*	65°	30 s	
Extension	72°	60 s/1 kb	
Final extension	72°	7 min	1
Hold	4°	Forever

*The optimal temperature for annealing depends on the Tm of the primers used.

d. Run the PCR products on a 1% (wt/vol) agarose gel (gel electrophoresis). Visualize the bands by Biorad GelDoc imager. Purify the PCR products of wild-type (control) and knockout PSC groups using a gel extraction kit as per manufacturers’ instruction, and sequence.
e. DNA sequence alignment tool (for instance, Multalign, NCBI Blastn, and Tide assay) can be used to analyze the indels in relevant sample groups (Supplementary Figures S1-S2).

**Note:** Tide assay was used to count indel frequency.

#### Checking “off-target effects” of a sgRNA

**Note:** Here, we checked the off-target effects of sgRNA by taking Mdm2 as the reference. The same procedure can be applied to check the off-target effects of all other sgRNAs.

##### Bioinformatic Analysis of Off-Target Effects

a. Check the sgRNA plus PAM sequence (23 nt) *via* the BLAST tool of NCBI (FASTA format).
b. Narrow down potential hits based on the BLAST sequence homology score Supplementary Protocol Section 2.6).
c. See a list of genome-wide *in silico* analyses of relevant sgRNAs targeting Stub1, Mdm2, and Yy1 genes in the Supplementary Material (Supplementary Protocol Section 2.6 and Supplementary Table S7).

##### PCR Primer Design, Amplification, and Sequencing

a. Design PCR primers of the genomic region of a certain gene partially overlapped by sgRNA. Design PCR primers and amplify products similarly as mentioned in *Determination of Indel Frequency of a gRNA*; see a list of PCR primers in the Supplementary Material (Supplementary Protocol Section 2.6.1 and Supplementary Table S8).
b. Subject the amplified PCR product to Sanger sequencing.

##### Verifying Sequenced Data With the Reference Genome

a. Use the BLASTN tool to compare sequencing data (FASTA format) with the reference genome (Supplementary Protocol Section 2.6.2).
b. Our analysis did not show any off-target effect of the relevant sgRNA (Supplementary Protocol Section 2.6.2).

**Troubleshooting**

**Problem 1**

Most of the PSCs die post-transfection.

**Potential solution**

- First, if PSCs are not healthy, use low-passage healthy cells.
- Second, check for mycoplasma or other bacterial contamination. Use only contamination-free cells.
- Third, dissolve PSCs into an Opti-MEM medium immediately after preparing the Lipofectamine-2000/plasmid mixture. Immediately, add the PSC-Opti-MEM mixture into Lipofectamine-2000-plasmid solution for no more than 5 min.
- The number of PSCs can be increased up to 5 × 10^5^.

**Problem 2**

Low PSC count after puromycin selection.

**Potential solution**

Optimize the dosage level of puromycin. Start puromycin selection from 0.5 up to 1 μg/ml.

**Problem 3**

Single PSCs form colonies very slowly.

**Potential solution**

Change the medium every day.

**Problem 4**

No obvious distinction between the PCR bands of wild-type/control and KO-targeted PSCs in gel electrophoresis.

**Potential solution**

It might happen due to the low efficiency of guider RNA. Design more guider RNAs (1–5).

**Problem 5**

No obvious bacterial colony on an agar plate.

**Potential solution**

Most possibly, digestion of plentiCRISPR V2 or pGL3-U6-sgRNA-PGK-puromycin is incomplete. Digest the plasmid again.

**Problem 6**

sgRNA with its PAM sequence overlaps with any genomic region during genome-wide off-target analysis in addition to its target site.

**Potential solution**

Design new sgRNA.

## Results and Discussion

Obtaining higher functional efficiency of genome editing is a crucial aspect of gene manipulation techniques in various mammalian cells as it is associated with laborious work, huge costs, and time consumption. Nonetheless, optimal genome-editing methods through CRISPR/Cas9 are insufficient for stem cell research, thereby affecting the translation of genome editing from the bench to bedside.

Lentiviruses, in common, are used for CRISPR/Cas9 delivery that confers risks of clinical utilization of CRISPRed cells. To brief the principle of our methodology, the backbone plasmid “pLentiCRISPR V2” does not contain any modification, rather to generate integration-free knockout PSCs, the pLentiCRISPR V2 constructs containing sgRNA were directly delivered into PSCs without any packaging or envelope plasmids through a modified liposome-mediated (lipofectamine) transfection method, which is called the single-cell transfection procedure in this study. In addition, this optimized protocol works with equal efficiency in a non-lentiviral vector system (e.g., pGL3-U6-sgRNA-PGK-puromycin and pST1374-NLS-Flag-Linker-Cas9) bearing different selectable markers. In this procedure, the liposome–DNA complex (liposome imparts positive charges onto DNA surface) was directly mixed with a single-cell suspension of PSCs for a shorter incubation period, resulting in enhanced fusion of DNA–liposome complexes with negatively charged plasma membranes of the cells, thereby allowing the quickest delivery of higher amounts of DNA into the cells. Only a small number of the cells (∼0.1%) survive after the antibiotic selection, thereby allowing cells to grow as single clones. Hence, it does not involve the laborious and time-consuming procedures of a lentivirus-mediated gene delivery system, which requires the passage of a lot of mutant cells as single clones into a 96-well plate. Every single cell forms a single colony (50–100 cells/colony) in 1 week that can be seen with naked eyes and can manually be picked up for further growth and knockout validation.

Our simplified protocol has some advantages, for example, i. non-viral delivery system (time-saving: 3–5 days): overall procedure does not need to generate viral particles to infect PSCs. ii. Minimal transfection time is only 24 h long (Figure 2). iii. Least duration of antibiotic selection (24 h): 24 h post-transfection, cells were selected with puromycin (single-vector system) or puromycin with blasticidin (two-vector system) for only 24 h (Figure 2), which save 5–7 days as the time is normally required for other methods of CRISPR/Cas9-assisted knockouts. iv. Using minimal numbers of the cell (∼8 × 10^3^ cells/cm^2^ or 2 × 10^4^) for transient transfection (1 μg) (Figure 2). v. No passaging of mutant cell pools into a single clone into 96-well plates (time-saving: 14–21 days): following antibiotic selection, only ∼0.1% cells survive, indicating no further requirement of expansion of mutant cells as a single cell rather every cell grows as a single-cell clone in the same plate. This is because of initial least cell density, quick transfection, and antibiotic selection that allow the surviving cells to grow as a single-cell clone (Figure 2). vi. Integration-free transgenes that encode for puromycin and Cas9: it is a non-viral delivery method. vii. Least chance of off-target effects due to the shortest duration of Cas9 expression and antibiotic selection. No off-target effects were observed following the knockout (Supplementary Material and Supplementary Protocol Section 2.6). viii. The highest percentage of cells displaying functional knockout efficiency: maximum five colonies of each sgRNA were randomly selected for Western blot validation of Mdm2, Stub1, and Yy1 expression. Based on protein expression analysis, an average of 90% colonies (CI = 95%, 79.5230%–100%) exhibited functional knockouts (Figure 3). The indel efficiency of sgRNAs was 67.12% (CI = 95% and 65.566%–75.108%) (Supplementary Figures S1, S2). ix. The modified transfection procedure is feasible and time-saving; hence, no need to use 293T cells for validating sgRNA efficiency as it is regularly done in other procedures (Sanjana et al., 2014; Shalem et al., 2014); instead, CRISPR constructs are directly delivered into PSCs (time-saving: 1–2 weeks). x. This protocol works efficiently with both lentiviral or non-lentiviral backbone, single- or two-vector system, and single or combinatorial drug selection. xi. Convenient, safe, inexpensive, and rapid protocol: overall procedure can be performed in 2 weeks (Figure 2).

The majority of the CRISPR/Cas9 knockout methodologies rely on viral delivery systems, for instance, lentivirus-mediated transduction of CRISPR/Cas9-gRNA constructs into cells, which poses questions for safe usage in clinical trials because the viral system might lead to unintended genome integration of Cas9 and selection markers (Vicente et al., 2021; Chakraborty, 2019). The viral delivery system requires additional time and high tighter viruses for successful knockout experiments (Sanjana et al., 2014; Shalem et al., 2014; Vicente et al., 2021; Jin et al., 2020). The potential risks of immunogenicity arising from the use of the viruses and Cas9 expression cannot be ruled out (Chakraborty, 2019). Though safety concern in viral approaches has sufficiently been resolved and improved, the off-target effects still exist due to the long-term exposure during the viral delivery method. The constitutive expression of Cas9 and prolonged antibiotic selection in the viral system might lead to higher levels of off-target mutagenesis, while certain cells possess a tendency to repress viral expressed proteins (Vicente et al., 2021; Jin et al., 2020; Ellis, 2005). To avoid these potential risks, our protocol is optimized with the quickest delivery and shortest exposure to both Cas9 and antibiotics. Prolonged antibiotic selection instead of 24 h resulted in the death of all PSCs, indicating that 72 hours post-transfection, the exogenous plasmids possibly degrade inside the cells, thereby allowing the development of transgene-free PSC colonies. Additionally, the PCR experiment with PSCs 10 days post-transfection does not reveal any detectable amplicon of transgene Cas9 (Figure 3). The most laborious step in viral delivery is to expand mutant cell pools into single clones (e.g., 96-well plate), which, depending on protocols, requires additional 2–3 weeks or more (Lino et al., 2018; Giuliano et al., 2019; Ishibashi et al., 2020; Lu et al., 2020). Some protocols also require both puromycin- and FACS-based selection, although this technique is praiseworthy since only cells with the highest transduction efficiency are selected (Lino et al., 2018; Giuliano et al., 2019; Ishibashi et al., 2020; Lu et al., 2020), yet these require sophisticated lab setup with flow cytometry and expertise, and are also very time-consuming and laborious. Our methodology in this context is very convenient and can be performed in a normal lab setup within the shortest period. Additionally, our protocol can be performed with a single- or two-vector system that can work regardless of the lentiviral or non-lentiviral backbone. Thus, our protocol provides an efficient and feasible CRISPR-assisted gene-editing method that can be completed in 2 weeks.

Our protocol has certain limitations; for instance, we took advantage of the inherent colony-forming capability of pluripotent stem cells, but the knockout of the target gene might enhance the differentiation, and in these cases, it needs prolonged time for single-cell expansion. Notably, this protocol cannot be applied to CRISPR/Cas9 knockout library-screening system.

## Data Availability

The original contributions presented in the study are included in the article/Supplementary Material, further inquiries can be directed to the corresponding author.
