# Supplementary figures and images for: Fast-Track and Integration-Free Method of Genome Editing by CRISPR/Cas9 in Murine Pluripotent Stem Cells
Source: Front Cell Dev Biol. 2022 Mar 18;10:819906. doi: 10.3389/fcell.2022.819906 (PMC8972586; doi:10.3389/fcell.2022.819906)

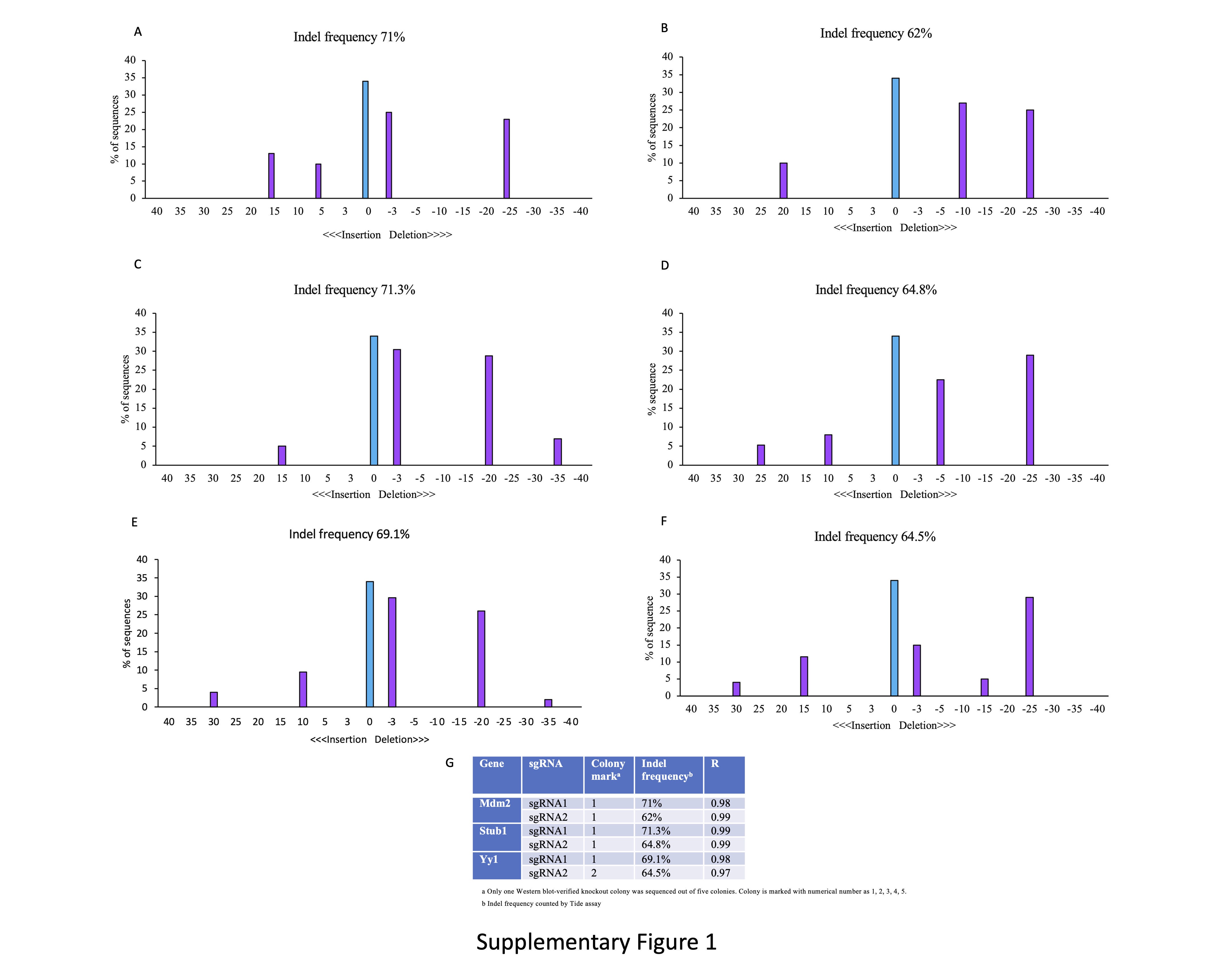

Supplement: Supplementary file 1 [file Image1.TIFF]

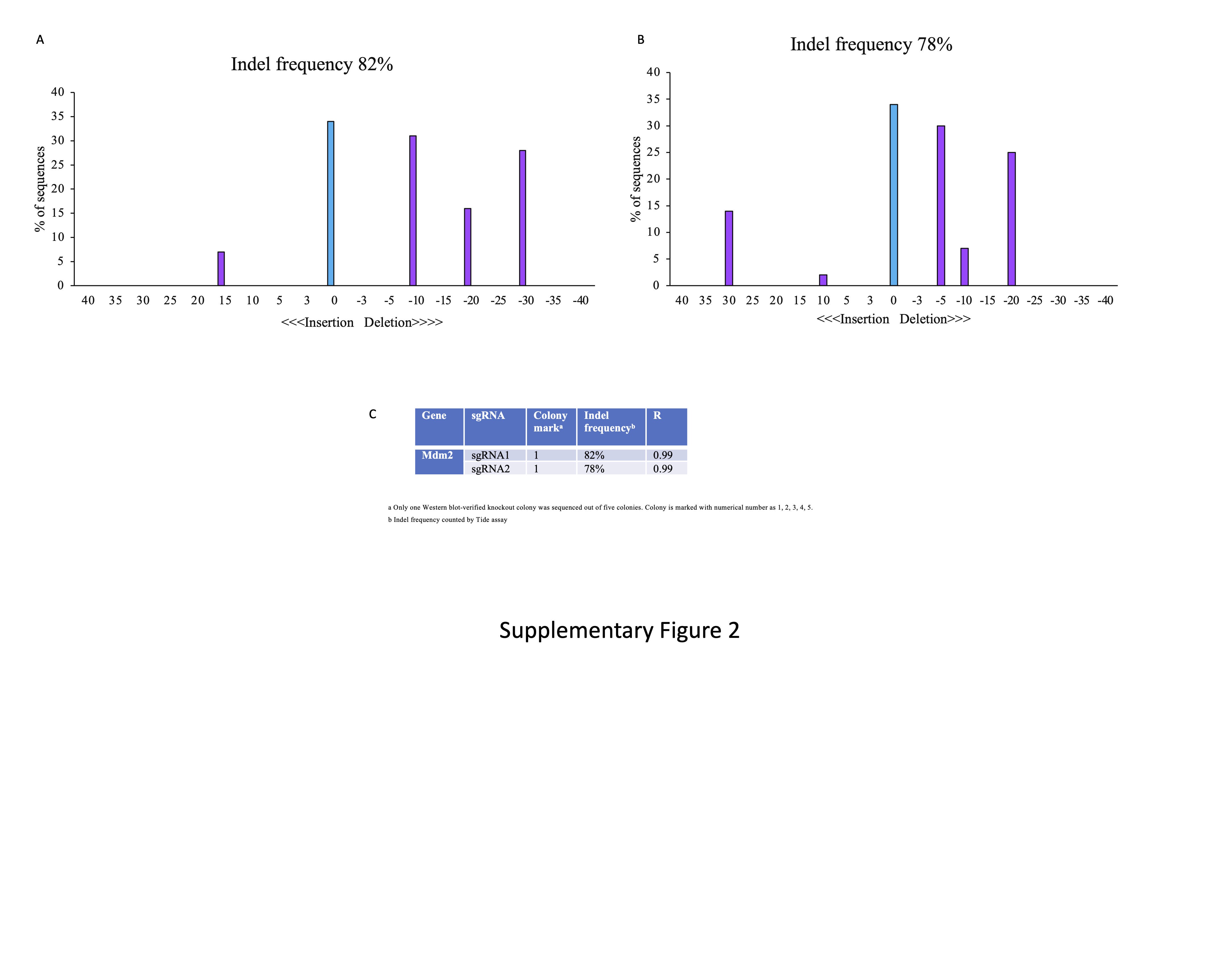

Supplement: Supplementary file 3 [file Image2.TIFF]
