# Supplementary material for: Fast-Track and Integration-Free Method of Genome Editing by CRISPR/Cas9 in Murine Pluripotent Stem Cells
Source: Front Cell Dev Biol. 2022 Mar 18;10:819906. doi: 10.3389/fcell.2022.819906 (PMC8972586; doi:10.3389/fcell.2022.819906)
Supplement: Supplementary file 2 [file DataSheet1.pdf]

Supplementary Information

1. Reagent formulation

Table 1: Key reagents and resources.

REAGENT or RESOURCE	SOURCE	IDENTIFIER
**Antibodies**		
Stub1 (WB 1:1000)	CST	Cat# 2080
Mdm2 (WB 1:1000)	CST	Cat# 27883-1-AP
Yy1 (1:1000)	CST	Cat# #46395
β-Actin (WB 1:1000)	CST	Cat# 3700
**Reagents**		
Puromycin	Sigma	Cat# P8833
BsmBI	NEB	Cat# R0580
Gibco™ DMEM, high glucose	Gibco	Cat#11584486
GlutaMAX™ Supplement	Gibco	Cat# 35050061
Trypsin-EDTA (0.25%)	Gibco	Cat# 25200056
ESGRO® Recombinant Mouse LIF Protein	Sigma	Cat# ESG1107
KnockOut™ Serum Replacement	Gibco	Cat# 10828028
Embryonic stem-cell FBS	Gibco	Cat# 16141079
D-PBS	Gibco	Cat# 14040141
2-Mercaptoethanol	Sigma	Cat# M7522
MEM Non-Essential Amino Acids Solution (100X)	Gibco	Cat# 11140050
Sodium Pyruvate (100 mM)	Gibco	Cat# 11360070
Penicillin-Streptomycin (10,000 U/mL)	Gibco	Cat# 15140122
EmbryoMax™ 0.1% Gelatin Solution	Millipore	Cat# ES-006
T4 DNA Ligase	Thermo	Cat# EL0011
RIPA buffer	Millipore	Cat# 20188
SDS-PAGE gel	Epizyme	Cat# PG112
Ampicillin	Beyotime Biotechnology	Cat# ST007

Tris-HCl	Sinopharm Chemical Reagent	Cat# 73509461
SDS	Amresco	Cat# 0227-1KG
Glycerol	Sinopharm Chemical Reagent	Cat# 10010618
Bromophenol blue	Sangon Biotech	Cat# A500922-0025
Agarose	Biowest	Cat# BY-R0100
Yeast extract	OXOID	Cat# LP0021
Tryptone	OXOID	Cat# LP0042
NaCl	Sangon Biotech	Cat# 7647-14-5
Agar powder	Solarbio	Cat# A8190
2 × Taq Plus Master Mix	Vazyme	Cat# P211-01
Calcium chloride dihydrate	Sigma	Cat# C7902
Lipofectamine™ 2000 Transfection Reagent	Thermo	Cat# 11668019
Opti-MEM™	Gibco	Cat# 31-985-062
**Experimental Model: Cell line**		
R1 embryonic stem cells (Mouse)	ATCC	SCRC-1011™
**Bacterial and virus strains**		
Trans5α Chemically Competent Cells	TransGen	Cat# 431675
**Critical commercial assays**		
GeneJet Gel Extraction Kit	Thermo	Cat# K0692
TIANamp Genomic DNA Kit	Tiangen	Cat# DP304-03
GeneJet Plasmid Miniprep Kit	Thermo	Cat# K0503
**Recombinant DNA**		
lentiCRISPRv2	Addgene	Cat# 52961
pGL3-U6-sgRNA-PGK-Puromycin	Addgene	Cat# 51133
pST1374-NLS-Flag-Linker-Cas9	Addgene	Cat# 44758
**Oligonucleotides**	Table 4-6	

1.1. Formulation of LB medium and agar plate:

i. Prepare LB buffer and autoclave at 121^0C for 15 min (Table 2). The LB buffer can be stored at 4^0 C.

Note: Add ampicillin to the LB buffer before use (100 µg/mL).

ii. To prepare agar plate, add 15g agar powder to 1LB and autoclave at 121^0 (Table 2). Cool adequately, add ampicillin (100 µg/mL) and keep it at room temperature to solidify. The LB buffer can be stored at 4^0 C for around 2 months.

Table 2. Formulation of LB medium and agar plate.

Reagent	Amount
Yeast extract	5g
Tryptone	10g
NaCl	10g
ddH2O	to 1L
Total	1L

Table 3. Formulation of 2X Laemili sample buffer.

Reagent	Amount to add	Final concentration (2X)
10% (w/v) SDS	4 mL	4%
Glycerol	2 mL	20%
1 M Tris-Cl (pH 6.8)	1.2 mL	120 mM
H_2O	2.8 mL	
Add bromophenol blue to a final concentration of 0.02% (w/v).		

Note: Store the 2X Laemmli sample buffer at room temperature.

2. Single Guider RNA (sgRNA) design and synthesis

Timing: 5 minutes

2.1. sgRNA design

Input target gene symbol/gene ID/transcript ID/genomic DNA sequence depending on the different CRISPR/Cas9 gRNA designing tools. For instances, CRISPick (Sanson et al., 2018;

Doench et al., 2016), Cas-OFFinder (Bae et al., 2014), CHOPCHOP (Labun et al., 2019), CRISPOR (Concordet & Haeussler, 2018), E-CRISPR (http://www.e-crisp.org/E-CRISP/index.html) etc. Here, we use CRISPRPick (Broad Institute GPP) tool to design our gRNA (Figure 1).

i. Use valid gene symbol (here, we proceed to knockout three genes, namely Mdm2, Stub1 and Yy1, and to simplify our description, we used Mdm2 as an example). Alternatively, use transcript ID or exon sequences of genomic DNA from Ensembl (https://ensembl.org/index.html).

CRITICAL: Selection of sgRNAs with higher specificity is recommended to minimize off-target effects. The specificity of a gRNA can be assessed by the BLAT tool in the UCSC genome browser (http://genome.ucsc.edu) or Blast tool (https://blast.ncbi.nlm.nih.gov).

ii. Check the specificity of a sgRNA using 20nt gRNA sequence plus PAM motif NGG (protospacer adjacent motif) by BLAT tool in the UCSC genome browser or Blast tool in NCBI. For example, Mdm2 sgRNA 1: 5′-TCGGAACAAGAGACTCTGGT (20nt) + 5′-TGG (PAM).

Note. Typically, design at least 2 sgRNAs that target two different gene coding regions of the desired gene.

2.2. sgRNA synthesis

2.2.1. One vector system (Lentiviral backbone): The following sgRNA oligonucleotide primers were designed for pLentiCRISPR V2 (Sanjana et al., 2014; Shalem et al., 2014) (Figure 1):

i. sgRNA oligonucleotide
sgRNA Forward: 5′-CACCGNNNNNNNNNNNNNNNNNNNN-3′
sgRNA Reverse: 5′-AAACNNNNNNNNNNNNNNNNNNNNC-3′

Note: The highlighted region (green) in the sgRNA oligonucleotide represents the 20nt sgRNA sequence of the forward primer and reverse-complementary sequence of this sgRNA in the

reverse primer. The sgRNA oligonucleotide must not contain NGG PAM. The overhangs: CACC and AAAC (black) following BsmBI digestion of pLentiCRISPR V2 are added to the 5′ end of each sgRNA primer respectively. G (red) after the overhang CACC is added to the 5′ region of forward sgRNA, and its complementary C (red) is added to the 3′ end of the reverse sgRNA primer because U6 promoter requires G to express the sgRNA. Copy and paste the sgRNAs of the desired gene (Mdm2, Stub1 and Yy1) separately into the highlighted region (Table 4).

Table 4. Lists of sgRNA oligonucleotides.

Oligonucleotides	Forward (Sequence 5'-3')	Reverse (Sequence 5'-3')
Mdm2-sgRNA 1	CACCG TCGGAACAAGAGACTCTGGT	AAAC ACCAGAGTCTCTTGTTCCGA C
Mdm2-sgRNA 2	CACCG CAGGCTCGGATCAAAGGACA	AAAC TGTCCTTTGATCCGAGCCTG C
Stub1-sgRNA 1	CACCG GAAGCGCTGGAACAGTATCG	AAAC CGATACTGTTCCAGCGCTTC C
Stub1 -sgRNA 2	CACCG GGAGATGGAGAGTTATGATG	AAAC CATCATAACTCTCCATCTCC C
Yy1-sgRNA 1	CACCG AGATATTGACCATGAAACAG	AAAC CTGTTTCATGGTCAATATCT C
Yy1-sgRNA 2	CACCG CGACCCGGGGAATAAGAAGT	AAAC ACTTCTTATTCCCCGGGTCG C

2.2.2. Two-vector system (non-lentiviral backbone): The sgRNA oligonucleotide primers are designed for pGL3-U6-sgRNA-PGK-puromycin in a two-vector system as follow (Figure 1):

i. sgRNA oligonucleotide (Two vector system)

sgRNA Forward: 5′- CCGGGNNNNNNNNNNNNNNNNNNNN-3′
sgRNA Reverse: 5′-AAACNNNNNNNNNNNNNNNNNNNNC-3′

Note: The highlighted region (green) in the sgRNA oligonucleotide represents the 20nt sgRNA sequence of the forward primer, and the reverse-complementary sequence of this sgRNA in the reverse primer. The sgRNA oligonucleotide should not contain NGG PAM. The overhangs: CCGG and AAAC (black) following Bsa I digestion of pGL3-U6-sgRNA-PGK-puromycin were added to the 5′ end of each sgRNA primer respectively. G (red) after the overhang CACC is added to the 5′ region of forward sgRNA, and its complementary C (red) is added to the 3′ end of the reverse sgRNA primer because U6 promoter requires G to express the sgRNA. Copy and paste the sgRNAs of the desired gene separately into the highlighted region (Table 5).

Table 5. Lists of sgRNA oligonucleotides.

Oligonucleotides	Forward (Sequence 5'-3')	Reverse (Sequence 5'-3')
Mdm2-sgRNA 1	CCGG TCGGAACAAGAGACTCTGGT	AAAC ACCAGAGTCTCTTGTTCCGA C
Mdm2-sgRNA 2	CCGG CAGGCTCGGATCAAAGGACA	AAAC TGTCCTTTGATCCGAGCCTG C
Stub1-sgRNA 1	CCGG GAAGCGCTGGAACAGTATCG	AAAC CGATACTGTTCCAGCGCTTC C
Stub1 -sgRNA 2	CCGG GGAGATGGAGAGTTATGATG	AAAC CATCATAACTCTCCATCTCC C
Yy1-sgRNA 1	CCGG AGATATTGACCATGAAACAG	AAAC CTGTTTCATGGTCAATATCT C
Yy1-sgRNA 2	CCGG CGACCCGGGGAATAAGAAGT	AAAC ACTTCTTATTCCCCGGGTCG C

2.3. Order the designed sgRNA oligonucleotides.

Note. In fact, de-salted standard oligos are sufficient for efficient cloning.

2.4. Preparation of single and two-vector constructs

Timing: 2 days

2.4.1. Annealing oligo pair:

a. Forward and reverse oligonucleotides of sgRNA were dissolved in ddH_2O and diluted to a final concentration of 10µM. The oligos were mixed as follow:

Component	Amount
Forward oligo (10µM)	1µl
Reverse oligo (10µM)	1µl
T4 ligation buffer (NEB) (10X)	1µl
ddH_2O	7µl
Total	10µl

Note: T4 ligation buffer (NEB) or any high salt appropriate buffer can be used.

b. The oligo mix can be annealed by heating at 95^0C for 5 minutes and cooling at room temperature (~25^0C).

2.4.2. Digestion of plentiCRISPRv2 or pGL3-U6-sgRNA-PGK-puromycin

a. Preparation of lentiCRISPRv2-BsmBI digestion mix:

**Component**	**Amount**
plentiCRISPRv2	1μg
BsmBI	0.5μl
NEbuffer3.1 (10X)	2μl
ddH_2O	16.5μl
Total	20μl

b. Preparation of pGL3-U6-sgRNA-PGK-puromycin-BsaI digestion mix:

**Component**	**Amount**
pGL3-U6-sgRNA-PGK-puromycin	1μg
BsaI	0.5μl
10X Buffer G	2μl
ddH_2O	16.5μl
Total	20μl

c. Keep the plentiCRISPRv2-BsmBI digestion mix at 55^0C for 1 hour or pGL3-U6-sgRNA-PGK-puromycin-BsaI digestion at 37^0C for 30 minutes.

d. Run the digestion mix on 1% (wt/vol) agarose gel (gel electrophoresis).

Note: Successful digestion will appear in two bands/fragments for plentiCRISPRv2 on the gel: 1. larger band (~13kb) and 2. shorter band (~2kb, filler piece).

e. Cut the gel containing larger fragments for plentiCRISPRv2 or a single fragment of pGL3-U6-sgRNA-PGK-puromycin and purify the digested plasmid using GeneJET gel extraction kit. Dissolve the extracted plasmid into 10μl ddH_2O.

2.4.3. Ligation of sgRNA oligonucleotides

a. Prepare the ligation reaction for each sgRNA as follows:

**Component**	**Amount** (μl)
BsmBI-digested plentiCRISPRv2 (from step 2.2)/<br>BsmBI-digested pGL3-U6-sgRNA-PGK-puromycin (from step 2.2)	2
sgRNA (from step 1)	8
10X T4 DNA ligase buffer	2
T4 DNA ligase	1
ddH2O	7
Total	20

b. Incubate the sgRNA-lentiCRISPRv2/ pGL3-U6-sgRNA-PGK-puromycin ligation reaction mix at 22^{0}C for 1 h.

c. Add the sgRNA- lentiCRISPRv2 ligation reaction mix/ pGL3-U6-sgRNA-PGK-puromycin ligation into an Eppendorf tube containing Trans5α chemically competent cells, incubate the mixture on ice for 30 min, followed by heat shock at 42^{0}C water bath for the 90s, and immediate re-incubation on ice for 5 min.

Note: To reduce the chances of potential homologous recombination, the transformation of lentiviral plasmids into recombination-deficient bacteria (e.g., Stbl3) is recommended.

d. Add 50-100μl LB to the ligation-bacteria mix from step 2.3.c and incubate the mixture at 37^{0}C for 45 minutes in a shaker.

e. Spread the mixture from step 2.3.d onto an agar LB dish (ampicillin) and incubate at 37^{0}C for 1 day.

f. Pick up ~3-5 bacterial colonies from step 2.3.e, grow single colony into liquid LB (100µg/mL ampicillin) and incubate bacterial suspension at 37^{0}C for 12-16 hours.

g. Extract plasmid DNA from the bacteria using GeneJET plasmid miniprep kit as per manufacturer’s instruction.

h. Confirm the gRNA sequence of plentiCRISPR-sgRNA plasmid/ pGL3-U6-sgRNA-PGK-puromycin-sgRNA plasmid by Sanger sequencing. Using U6 primer sequence each bacterial colony.

Note: The 20bp gRNA sequence should be placed between the U6 promoter and the remainder of the gRNA scaffold in the plentiCRISPR v2 construct.

2.5. Functional validation of knockout cells

2.5.1. Determination of indel frequency of a sgRNA

Table 6. List of PCR primers.

Oligonucleotides	Forward (Sequence 5'-3')	Reverse (Sequence 5'-3')	Amplicon size
Amplicon-Mdm2-gRNA1	ATGTGCAATACCAACATGTCTG	GTCTCACTAATGGATCTCCTTCTAG	550bp
Amplicon-Mdm2-gRNA2	GAAGAGAAACCTTCATCTTCTG	GTTCTCACGAAGGGTCCAG	555bp
Amplicon-Stub1-gRNA1	ATCTCCAGTTCCTCTATTCCAG	TGAGATATGAATGCAGCTCAC	550bp
Amplicon-Stub1-gRNA2	TGTACTACACTAACCGGGC	TGACCAGGAAATCACAGAGC	550bp
Amplicon-Yy1-gRNA1	GCTGCTTCTGCTAGGCTAAC	GCTGCCCTTCACACATCAAC	554bp
Amplicon-Yy1-gRNA2	ATCCTGGTGCAGACGCGCGAG	GCTTCCGCTCGCACAAACTA	560bp
Amplicon-Cas9	ACAAGTTCATCAAGCCCATC	GAATCTGCTGTTTCCCCTG	269bp

2.6. Verifying the 'Off-target Effects' of a sgRNA

Table 7. Genome-wide *in-silico* analysis of sgRNA plus PAM sequence.

sgRNA Name	Overlapping with PAM sequence (Entire mouse genome)	Full coverage (Entire mouse genome)
Mdm2-sgRNA 1	No	Mdm2 (only sgRNA target site)
Mdm2-sgRNA 2	No	Mdm2 (only sgRNA target site)
Stub1-sgRNA 1	No	Stub1 (only sgRNA target site)
Stub1 -sgRNA 2	No	Stub1 (only sgRNA target site)
Yy1-sgRNA 1	No	Yy1 (only sgRNA target site)
Yy1-sgRNA 2	No	Yy1 (only sgRNA target site)

I. Mdm2 (Partial coverage of sgRNA sequence +No coverage of PAM sequence) (partial coverage)

Range 2: 93600689 to 93600704

Score	Expect	Identities	Gaps	Strand
32.2 bits(16)	3.3	16/16(100%)	0/16(0%)	Plus/Minus

Features: 18974 bp at 5' side: netrin-4 precursor80848 bp at 3' side: ubiquitin carboxyl-terminal hydrolase 44 isoform x1

Query 6 ACAAGAGACTCTGGTT 21

Sbjct 93600704 ACAAGAGACTCTGGTT 93600689

II. Mdm2 (Partial coverage of sgRNA sequence +No coverage of PAM sequence) (partial coverage)

Range 3: 19728780 to 19728794

Score	Expect	Identities	Gaps	Strand
30.2 bits(15)	13	15/15(100%)	0/15(0%)	Plus/Plus

Features: 2012 bp at 5' side: solute carrier family 35 member d3 isoform x17878 bp at 3' side: peroxisomal biogenesis factor 7 isoform 2

Query 8 AAGAGACTCTGGTTG 22

Sbjct 19728780 AAGAGACTCTGGTTG 19728794

III. Mdm2 (Partial coverage of sgRNA sequence +No coverage of PAM sequence) (partial coverage)

Range 4: 54329247 to 54329261

Score	Expect	Identities	Gaps	Strand
30.2 bits(15)	13	15/15(100%)	0/15(0%)	Plus/Plus

Features:
378127 bp at 5' side: mannosyl-oligosaccharide 1,2-alpha-mannosidase ia1564420 bp at 3' side: protein broad-minded

Query 9 AGAGACTCTGGTTGG 23

Sbjct 54329247 AGAGACTCTGGTTGG 54329261

2.6.1. Design primers of Mdm2:

Table 8. List of PCR primers.

**Oligonucleotides**	**Forward (Sequence 5'-3')**	**Reverse (Sequence 5'-3')**	**Amplicon size**
I. Mdm2 partial sequence homology gRNA1_ ubiquitin carboxyl-terminal hydrolase 44 isoform x1	GGGCAGCAAAGATATTAGTGG	GGATAGGATTTGGTAAAGTGGG	404bp
II. Mdm2 partial sequence homology gRNA1_ ubiquitin carboxyl peroxisomal biogenesis factor 7 isoform 2	AAACCCTCCTTGCCTCCATC	TCTTTCTTGTTCAGCCCCTTC	510bp
III. Mdm2 partial sequence homology gRNA1_ protein broad-minded biogenesis factor 7 isoform 2	CATTACAGCTCGAAGCAGAC	TCCGATGATGACCTTCTTCC	488bp

2.6.2. Analysis of sequencing results by Blast

I. Mdm2 partial sequence homology gRNA1_ ubiquitin carboxyl-terminal hydrolase 44 isoform x1

Score	Expect	Identities	Gaps	Strand
712 bits(385)	0.0	385/385(100%)	0/385(0%)	Plus/Minus

```
Query  32     GGGCAGCAAAGATATTAGTGGGCAGAAGCTTTGCAGCTCAGCACAGCCATGGCTGAAGAG  91
              ||||||||||||||||||||||||||||||||||||||||||||||||||||||||||||
Sbjct  19363  GGGCAGCAAAGATATTAGTGGGCAGAAGCTTTGCAGCTCAGCACAGCCATGGCTGAAGAG  19304

Query  92     GACAGTGTGACATGCCAGACAAGAGACTCTGGTTCCTGCCAGGCAACAGCAATAACGTCT  151
              ||||||||||||||||||||||||||||||||||||||||||||||||||||||||||||
Sbjct  19303  GACAGTGTGACATGCCAGACAAGAGACTCTGGTTCCTGCCAGGCAACAGCAATAACGTCT  19244

Query  152    GGCTATCGCACTCCAAATAAAATCTATAATGAAATGGTCAGAGGGCAAAGACCAGGGCTA  211
              ||||||||||||||||||||||||||||||||||||||||||||||||||||||||||||
Sbjct  19243  GGCTATCGCACTCCAAATAAAATCTATAATGAAATGGTCAGAGGGCAAAGACCAGGGCTA  19184

Query  212    ACGCAGTTCCTGAAGATGCCTGATGTCCCACAAAGTAAAGGCAGAGTAGAGACACTGGAT  271
              ||||||||||||||||||||||||||||||||||||||||||||||||||||||||||||
Sbjct  19183  ACGCAGTTCCTGAAGATGCCTGATGTCCCACAAAGTAAAGGCAGAGTAGAGACACTGGAT  19124

Query  272    AAAGGGGGACATAGTTTCCGTGATAGAGATGCAGTGCTGGGGGAAAGACTTCCAGCCAAC  331
              ||||||||||||||||||||||||||||||||||||||||||||||||||||||||||||
Sbjct  19123  AAAGGGGGACATAGTTTCCGTGATAGAGATGCAGTGCTGGGGGAAAGACTTCCAGCCAAC  19064

Query  332    AGCCTTGGGAAAAGGAGAGCCTGTTGAGTTCAGGGAGACAAATATTTACGGAGCGAGCGT  391
              ||||||||||||||||||||||||||||||||||||||||||||||||||||||||||||
Sbjct  19063  AGCCTTGGGAAAAGGAGAGCCTGTTGAGTTCAGGGAGACAAATATTTACGGAGCGAGCGT  19004

Query  392    GAGACGGGAGAATGAGTAGCTTCCC  416
              |||||||||||||||||||||||||
Sbjct  19003  GAGACGGGAGAATGAGTAGCTTCCC  18979
```

II. Mdm2 partial sequence homology gRNA1_ ubiquitin carboxyl peroxisomal biogenesis factor 7 isoform 2

Score	Expect	Identities	Gaps	Strand
856 bits(463)	0.0	463/463(100%)	0/463(0%)	Plus/Minus

```
Query  1      CGCGCTCGCCTTCTGGATCAGCACCAGGTAGGCTGCGTGCACCAATACAGCCAGCACGCC  60
              ||||||||||||||||||||||||||||||||||||||||||||||||||||||||||||
Sbjct  22670  CGCGCTCGCCTTCTGGATCAGCACCAGGTAGGCTGCGTGCACCAATACAGCCAGCACGCC  22611

Query  61     CGTTACGTACCCAATGGGGTCGCCCGTCAGGTCGCCAGCTCCTGAGCGCACCAAGACGGC  120
              ||||||||||||||||||||||||||||||||||||||||||||||||||||||||||||
Sbjct  22610  CGTTACGTACCCAATGGGGTCGCCCGTCAGGTCGCCAGCTCCTGAGCGCACCAAGACGGC  22551

Query  121    AGAGCCAGCGCGGGAAAGAAAAAGAGAAGACACAGGGTTGGGTATCCACTCCAGGAAGCA  180
              ||||||||||||||||||||||||||||||||||||||||||||||||||||||||||||
Sbjct  22550  AGAGCCAGCGCGGGAAAGAAAAAGAGAAGACACAGGGTTGGGTATCCACTCCAGGAAGCA  22491

Query  181    TGTCGCCTTGGGGGGAAAACCCTTAGCATCCCAATTCTGCCCACGAATTTTCCGAGTGCC  240
              ||||||||||||||||||||||||||||||||||||||||||||||||||||||||||||
Sbjct  22490  TGTCGCCTTGGGGGGAAAACCCTTAGCATCCCAATTCTGCCCACGAATTTTCCGAGTGCC  22431

Query  241    CAACAGAAACCCTCCTTGCCTCCATCTCTGTGTCAAAATGCGACCGTCAAGCCGAGCTAA  300
              ||||||||||||||||||||||||||||||||||||||||||||||||||||||||||||
Sbjct  22430  CAACAGAAACCCTCCTTGCCTCCATCTCTGTGTCAAAATGCGACCGTCAAGCCGAGCTAA  22371

Query  301    TGTACCCGGTCTTCCTCTAGCCAGGGCGAAGGCCAGGGGGAGATCAGATGCTCTCTGGGT  360
              ||||||||||||||||||||||||||||||||||||||||||||||||||||||||||||
Sbjct  22370  TGTACCCGGTCTTCCTCTAGCCAGGGCGAAGGCCAGGGGGAGATCAGATGCTCTCTGGGT  22311

Query  361    AGGCTCCTTGGGGTCCCTGAAGCCTGCACGGAGGCCACCTGGGAGGGTGGTTCCCGCCCC  420
              ||||||||||||||||||||||||||||||||||||||||||||||||||||||||||||
Sbjct  22310  AGGCTCCTTGGGGTCCCTGAAGCCTGCACGGAGGCCACCTGGGAGGGTGGTTCCCGCCCC  22251

Query  421    GTGGGCACCGGAAGGGACAGGGAACCCCAGCTAGCGGCCTCAA  463
              |||||||||||||||||||||||||||||||||||||||||||
Sbjct  22250  GTGGGCACCGGAAGGGACAGGGAACCCCAGCTAGCGGCCTCAA  22208
```

III. Mdm2 (Partial coverage of sgRNA sequence +No coverage of PAM sequence) (partial coverage)

Score	Expect	Identities	Gaps	Strand
963 bits(521)	0.0	521/521(100%)	0/521(0%)	Plus/Minus

```
Query  1       AAACATTACAGCTCGAAGCAGACAGCATCTCCTCATAGAGGAGGAAGAAGTTTTGCAGTC  60
               ||||||||||||||||||||||||||||||||||||||||||||||||||||||||||||
Sbjct  157940  AAACATTACAGCTCGAAGCAGACAGCATCTCCTCATAGAGGAGGAAGAAGTTTTGCAGTC  157881

Query  61      AAATCAAATCATAATCAAGAGGTCACTGGTAGACGGTTCTATTATGAAAATGATTTTGCA  120
               ||||||||||||||||||||||||||||||||||||||||||||||||||||||||||||
Sbjct  157880  AAATCAAATCATAATCAAGAGGTCACTGGTAGACGGTTCTATTATGAAAATGATTTTGCA  157821

Query  121     ACTTTATGAATTTCAGAAACAATAGATTCCTTGAGAAAAAGAAAAAGACAGGCAGAATCT  180
               ||||||||||||||||||||||||||||||||||||||||||||||||||||||||||||
Sbjct  157820  ACTTTATGAATTTCAGAAACAATAGATTCCTTGAGAAAAAGAAAAAGACAGGCAGAATCT  157761

Query  181     GTGGATCTACTAGCTGAATAAGGAATTGAGGTCCACCTAGGCTTATGGCACTAAACATAA  240
               ||||||||||||||||||||||||||||||||||||||||||||||||||||||||||||
Sbjct  157760  GTGGATCTACTAGCTGAATAAGGAATTGAGGTCCACCTAGGCTTATGGCACTAAACATAA  157701

Query  241     AAGACTGCAAAACAATTGTAGAACTTAGGAGAATCATAGACGTTTGCCAATCAATAAGAG  300
               ||||||||||||||||||||||||||||||||||||||||||||||||||||||||||||
Sbjct  157700  AAGACTGCAAAACAATTGTAGAACTTAGGAGAATCATAGACGTTTGCCAATCAATAAGAG  157641

Query  301     TTCATCATATGTCTGATTACCAGAAAAGGAATTATTAAGGTATGCAGTGAGGGAACGAAG  360
               ||||||||||||||||||||||||||||||||||||||||||||||||||||||||||||
Sbjct  157640  TTCATCATATGTCTGATTACCAGAAAAGGAATTATTAAGGTATGCAGTGAGGGAACGAAG  157581

Query  361     CAGAGTAAAATAGAAAATGTCTTTCATTTCTCTTTGCCATCAATTTCCTTCTTCTGTTCA  420
               ||||||||||||||||||||||||||||||||||||||||||||||||||||||||||||
Sbjct  157580  CAGAGTAAAATAGAAAATGTCTTTCATTTCTCTTTGCCATCAATTTCCTTCTTCTGTTCA  157521

Query  421     CGGAGTATAGGGAAAGGATGAGCCTCGGTGTTGAAGATCCAGTGTTCTAGTGGAAGAAGG  480
               ||||||||||||||||||||||||||||||||||||||||||||||||||||||||||||
Sbjct  157520  CGGAGTATAGGGAAAGGATGAGCCTCGGTGTTGAAGATCCAGTGTTCTAGTGGAAGAAGG  157461

Query  481     TCATCATCGGAAAATATCAAGTACAAATACCTGGAGGGAGG  521
               |||||||||||||||||||||||||||||||||||||||||
Sbjct  157460  TCATCATCGGAAAATATCAAGTACAAATACCTGGAGGGAGG  157420
```

References

Bae S., Park J., & Kim J.-S. Cas-OFFinder: A fast and versatile algorithm that searches for potential off-target sites of Cas9 RNA-guided endonucleases. Bioinformatics 30, 1473-1475 (2014).

Concordet JP, Haeussler M. CRISPOR: intuitive guide selection for CRISPR/Cas9 genome editing experiments and screens. Nucleic Acids Res. 2018 Jul 2;46(W1):W242-W245. doi: 10.1093/nar/gky354. PMID: 29762716; PMCID: PMC6030908.

Doench JG, Fusi N, Sullender M, Hegde M, Vaimberg EW, Donovan KF, Smith I, Tothova Z, Wilen C, Orchard R, Virgin HW, Listgarten J, Root DE. Optimized sgRNA design to maximize activity and minimize off-target effects of CRISPR-Cas9. Nat Biotechnol. 2016 Feb;34(2):184-191. doi: 10.1038/nbt.3437. Epub 2016 Jan 18. PMID: 26780180; PMCID: PMC4744125.

Labun K, Montague TG, Krause M, Torres Cleuren YN, Tjeldnes H, Valen E. CHOPCHOP v3: expanding the CRISPR web toolbox beyond genome editing. Nucleic Acids Res. 2019 Jul 2;47(W1):W171-W174. doi: 10.1093/nar/gkz365. PMID: 31106371; PMCID: PMC6602426.
Sanjana NE, Shalem O, Zhang F. Improved vectors and genome-wide libraries for CRISPR screening. Nat Methods. 2014;11(8):783-784. doi:10.1038/nmeth.3047

Sanson KR, Hanna RE, Hegde M, Donovan KF, Strand C, Sullender ME, Vaimberg EW, Goodale A, Root DE, Piccioni F, Doench JG. Optimized libraries for CRISPR-Cas9 genetic screens with multiple modalities. Nat Commun. 2018 Dec 21;9(1):5416. doi: 10.1038/s41467-018-07901-8. PMID: 30575746; PMCID: PMC6303322.

Shalem O, Sanjana NE, Hartenian E, Shi X, Scott DA, Mikkelson T, Heckl D, Ebert BL, Root DE, Doench JG, Zhang F. Genome-scale CRISPR-Cas9 knockout screening in human cells. Science. 2014 Jan 3;343(6166):84-87. doi: 10.1126/science.1247005. Epub 2013 Dec 12. PMID: 24336571; PMCID: PMC4089965.
